# The Photophone—Transmission of Sound by Light

**Published:** 1881-04

**Authors:** 


### THE PHOTOPHONE—TRANSMISSION OF SOUND BY LIGHT.

Dr. A. Graham Bell, of Washington, D. C., inventor of the Bell Telephone, gave an account yesterday, in Hopkins Hall, Johns Hopkins University, of his new invention, the photophone. Dr. Bell is well known as a scientist, not only in this country but throughout Europe, and last year received the Volta prize of the French government for his researches in electricity. The remarkable instrument known as a photophone he invented about a year ago, and some additions which have been made to it were first made known on Thursday afternoon, April 21, 1881, before the National Academy of Sciences, in Washington. The inventor explained that what he had in view in this matter of the photophone was the substitution of a ray of light between telephonic stations in place of wire for the transmission, or rather the reproduction of sound. A strong beam of light sent from one telephonic station to another enables the sound to travel along it as along a wire. Mr. Sumner Taintor was associated with Dr. Bell in making the experiments which led to successful results. Dr. Bell went back to 1817, when Berzelius discovered tellurium, and later silenium, and traced the history of these metals in connection with telegraphy up to the present time, giving the part they had played in the construction of the photophone. In 1873 Willougby Smith discovered that the silenium offered as much resistance as a telegraph wire reaching from the earth to the moon, but this resistance was found to be very variable and affected by light. Silenium was used by the experimenters in order to have the rays of light vary. The metal was put into cells, and these connected with a telephonic and galvanic battery. A beam of light was thrown through an aperture, and a successive trembling of the rays of light, given at the rate of 600 to 1,000 per second by the turning of a wheel, produced the sound. The greatest distance to which thus sound has been transmitted is 1¼ miles. Cylinders of silenium were made later to be used in connection with parabolic mirrors. The whole matter is accomplished by the variation of the intensity of a ray of light. A transmitter was described, made of a thin mirror, which, when spoken against, by it disturbance caused a variation in the beams of light which it reflected. These rays, collected in a parabolic reflector, reproduced the words spoken against the mirror. This had been done successfully at a distance of 900 feet. An interesting account was given of the putting of various substances to intercept the ray of light. An opaque ebonite substance would not stop the ray of light, though the intervention of a hand would. An invisable beam came through the ebonite and produced sound. It was shown that different substances gave off different degrees of sound when a ray of light was thrown on them, and an instrument for measuring such differences of sound was described. Lampblack was found to answer in place of silenium for producing electrical disturbance. By means of these new discoveries the speaker stated that he anticipated that what he called and explained as the spectrophone would be an adjunct to the spectroscope.—*Balto. Sun.*

